# A machine learning approach to identify risk factors for running-related injuries: study protocol for a prospective longitudinal cohort trial

**DOI:** 10.1186/s13102-022-00426-0

**Published:** 2022-04-26

**Authors:** A. L. Rahlf, T. Hoenig, J. Stürznickel, K. Cremans, D. Fohrmann, A. Sanchez-Alvarado, T. Rolvien, K. Hollander

**Affiliations:** 1 Department of Sports Science, Institute of Health, Nutrition and Sports Science, Europa-Universität Flensburg, Campusallee 2, 24943 Flensburg, Germany; 2 Department of Trauma and Orthopaedic Surgery, University Medical Center Hamburg-Eppendorf, Martinistrasse 52, 20246 Hamburg, Germany; 3 Department of Osteology and Biomechanics, University Medical Center Hamburg-Eppendorf, Hamburg, Germany; 4 Department of Mechanical Engineering, Institute of Modelling and High-Performance Computing, Niederrhein University of Applied Sciences, Reinarzstraße 49, 47805 Krefeld, Germany; 5 Institute of Interdisciplinary Exercise Science and Sports Medicine, MSH Medical School Hamburg, Am Kaiserkai 1, 20457 Hamburg, Germany; 6 Department of Sports and Exercise Medicine, Institute of Human Movement Science, University of Hamburg, Turmweg 2, 20148 Hamburg, Germany

**Keywords:** Sports injuries, Risk factor analysis, Machine learning models

## Abstract

**Background:**

Running is a very popular sport among both recreational and competitive athletes. However, participating in running is associated with a comparably high risk of sustaining an exercise-related injury. Due to the often multifactorial and individual reasons for running injuries, a shift in thinking is required to account for the dynamic process of the various risk factors. Therefore, a machine learning approach will be used to comprehensively analyze biomechanical, biological, and loading parameters in order to identify risk factors and to detect risk patterns in runners.

**Methods:**

The prospective longitudinal cohort study will include competitive adult athletes, running at least 20 km per week and being free of injuries three months before the start of the study. At baseline and the end of the study period, subjective questionnaires (demographics, injury history, sports participation, menstruation, medication, psychology), biomechanical measures (e.g., stride length, cadence, kinematics, kinetics, tibial shock, and tibial acceleration) and a medical examination (BMI, laboratory: blood count, creatinine, calcium, phosphate, parathyroid hormone, vitamin D, osteocalcin, bone-specific alkaline phosphatase, DPD cross-links) will be performed. During the study period (one season), continuous data collection will be performed for biomechanical parameters, injuries, internal and external load. Statistical analysis of the data is performed using machine learning (ML) methods. For this purpose, the correlation of the collected data to possible injuries is automatically learned by an ML model and from this, a ranking of the risk factors can be determined with the help of sensitivity analysis methods.

**Discussion:**

To achieve a comprehensive risk reduction of injuries in runners, a multifactorial and individual approach and analysis is necessary. Recently, the use of ML processes for the analysis of risk factors in sports was discussed and positive results have been published. This study will be the first prospective longitudinal cohort study in runners to investigate the association of biomechanical, bone health, and loading parameters as well as injuries via ML models. The results may help to predict the risk of sustaining an injury and give way for new analysis methods that may also be transferred to other sports.

*Trial registration*: DRKS00026904 (German Clinical Trial Register DKRS), date of registration 18.10.2021.

**Supplementary Information:**

The online version contains supplementary material available at 10.1186/s13102-022-00426-0.

## Background

Running is one of the most popular sports worldwide. Despite strong evidence for the health benefits, the incidence of musculoskeletal overuse injuries remains high. In a recently published systematic review, almost half of the 22,823 runners sustained an injury during the respective observation period [1]. Depending on the study design and investigated cohort the injury rates vary between 19–79% [2, 3]. For instance, long-distance runners but also novice runners are more susceptible to sustain an injury compared to short-distance runners, and recreational runners [4, 5]. No difference, however, was found for the overall injury rate in females (20.8 injuries per 100 runners) and male runners (20.4 injuries per 100 runners) [6].

Many studies have methodological weaknesses, e.g., retrospective data collection, lack of load monitoring, lack of multivariable analysis of external and internal risk factors, or diagnosis based on patient self-report [3, 7]. Furthermore, the multifactorial influence of external and internal risk factors on musculoskeletal injuries—e.g., bone stress injuries, tendinopathies, and muscle injuries—has not yet been sufficiently clarified [8, 9]. Despite the multifactorial nature, the underlying etiology of overuse injuries can be explained by an imbalance between load and recovery [7, 10, 11]. Thus, runners with rapidly increased training volume as well as runners with too low training intensity showed an increased risk of injuries [12, 13]. Based on this information, the identification of risk factors for the development of running-related injury should occur simultaneously with objective training load monitoring.

In addition to loading parameters, internal (e.g., anatomy, biomechanics, musculoskeletal tissue quality) and external characteristics (e.g., environment, surface, footwear) are discussed as important risk factors [9, 14]. Since running injuries are predominantly attributable to overuse [1, 15], the combined analysis of bone and muscle status, biomechanics and the individual running technique represent an important approach to identify risk factors for these injuries. In this context, for example, vitamin D, bone density and microarchitecture [16, 17], ground reaction forces, load rates, foot strike, and cadence are discussed as important parameters [8, 16–21]. Current research in the field of sports injuries indicates that a shift in thinking from single risk factors to individual injury patterns that are dynamically influenced by a variety of mediators is necessary [22].

To account for the individual approach and the high variation of responsible mediators, different machine learning (ML) models have been used in the past to analyze risk factors in sports [23, 24]. ML models can learn the relationship between input and output variables solely from large amounts of example data with some kind of optimization algorithm. This enables the prediction of future outcomes from new input data without the need for manually programmed functions [25]. Some of these predictive modelling techniques used in association with sports injury prediction and prevention are for example Artificial Neural Networks, Support Vector Machines, and Random Forests [23]. Especially in the analysis of risk factors and the prediction of team sports injuries [23] or neuromuscular and musculoskeletal pathologies [26], promising results have been presented utilizing ML models in previous studies.

In contrast to the methods mentioned so far, a new method called Deep Gaussian Covariance Network (DGCN) [27] is used as the ML model. This represents a unique combination of neural networks and Gaussian processes $({\mathcal{G}\mathcal{P}})$ [28]. Gaussian processes are probabilistic ML models and thus offer the advantage of predicting model uncertainty. This means that the prediction of possible injuries can always be accompanied by a prediction of the certainty of the model.

The objective of the present study is to (a) prospectively monitor the injury incidence and characteristics, (b) determine internal and external risk factors and their interaction, and (c) evaluate the association of risk factors via machine learning processing to predict the risk of injuries in runners.

## Methods

### Study design

**Fig. 1:**
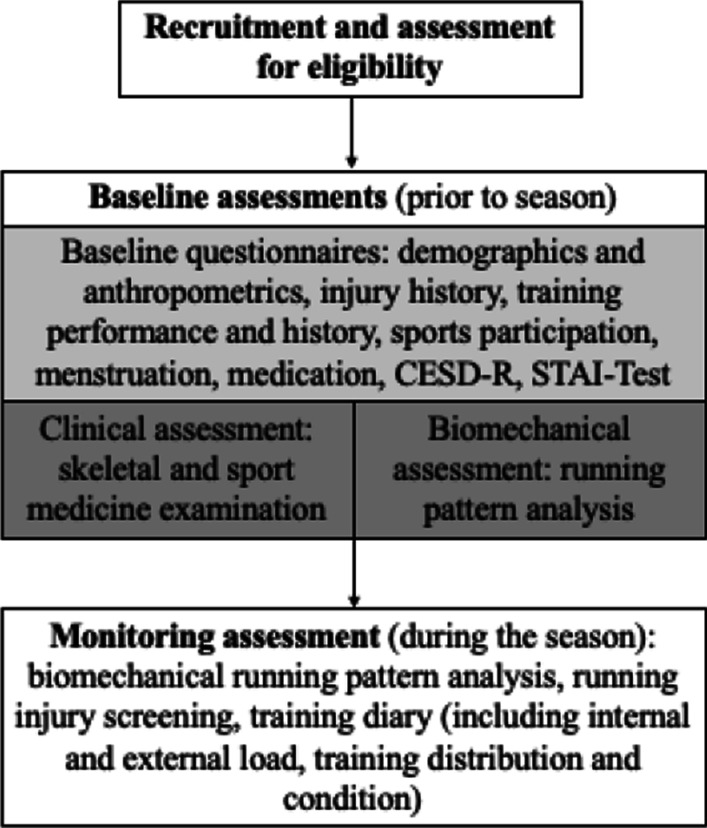
Study flow

The athlete’s injury monitoring and determination of the internal and external risk variables will be conducted in a prospective observational cohort study. During a season (approximately ten months), 120 athletes will be monitored for injuries, internal and external load, and biomechanical running parameters (Fig. 1). The study will be performed following the Good Clinical Practice guidelines [29] and in line with the Declaration of Helsinki. The present study protocol is prepared according to the Standard Protocol Items: Recommendations for Interventional Trials (SPIRIT) 2013 Statement [30].

### Ethical standard

Ethical approval was obtained through the local ethics committee of the chamber of Physicians Hamburg (reference no.: 2021-10458BO-ff). All potential subjects need to give their informed consent before study enrolment. Based on the study description, participants are informed that they can withdraw their consent to participate at any time. This does not result in any disadvantage for the subject and the data are excluded from the analysis and deleted.

### Participants

Potential participants will be recruited from running clubs and associations with competitive runners. Additionally, a call for participation will be made through social media and local running stores. The study will include female and male runners aged 18 years and older with a weekly training volume of 20 km or more (annual average at the time of study inclusion). Competitive runners are defined by estimated participation in at least one competition/race during the study period. Athletes can only be included in the study if they have been free of injury for at least 3 months.

### Assessment procedure

The study procedure provides a baseline assessment including (1) biomechanical measures, (2) subjective questionnaires, and (3) a clinical musculoskeletal examination at the beginning and the end of the study period (season of 10 months). In addition, biomechanical data on running parameters, internal and external load, and injuries will be collected continuously throughout the season. The tests will be conducted in three different locations. The biomechanical testing will be performed on a university outdoor running track and in a biomechanics laboratory. The clinical testing will take place at the specialized outpatient clinic for musculoskeletal disorders. All patients will undergo the medical examination and the biomechanical measurements within 7 days. Before the biomechanical measurement, a written informed consent will be required from all participants, and everyone will obtain a standardized study description including information about the assessment procedure.

### Baseline questionnaires

Before performing the biomechanical assessments, the athletes have to complete a baseline questionnaire including items about demographics and anthropometrics (weight, height, body mass index, age, sex), injury history, running performance, and history (weekly training volume, mean running speed, competition distance, personal bests, change in training volume and intensity during the past 12 months), sports participation in addition to running, menstruation, and medication. The baseline questionnaire is based on a survey published by Tenforde and colleagues identifying risk factors for running-related bone stress injuries (Tenforde et al., 2013). Furthermore, the athletes will be asked to answer questionnaires about their psychological health. For this purpose, standardized questionnaires for depression (CESD-R: Center for Epidemiologic Studies Depression Scale-Revised) and anxiety (STAI-Test: State-Trait-Anxiety Inventory) will be used [31–34]. The survey instrument CESD-R is freely available. The STAI- Test requires a license that can be purchased on the homepage www.mindgarden.com.

### Biomechanical baseline assessments

To record the individual running patterns, all participants will complete a baseline reference run on a running track and a biomechanics laboratory assessment.

#### Running track assessment

The baseline reference run will be measured by wireless inertial measurement units (IMUs) and magnetic gates integrated into the track (SmarTracks Diagnostics DX3.5, Humotion GmbH, Muenster, Germany). The magnetic gates are placed below the 400 m running track at a distance of 50 m as well as every 10 m in the section of the 100 m home straight. Each of the three running units starts one meter in front of a magnetic gate of the SmarTracks System which is considered as starting line.

In combination with the IMU, the system can collect spatiotemporal parameters about e.g. the distance, duration, and intervals [35]. Further, the integrated technology of the sensor can detect various characteristics of the running patterns by acceleration and rotation signals. We primarily focus on the parameters stride length, cadence, ground contact time, tibial shock, and tibial acceleration [36–39]. The IMUs have a size of 50 × 10 mm and will be fastened around the waist (sensor on fifth lumbar spine – L5) with the help of an elastic waist belt. In addition to the sensor placed on L5 (500 Hz), one sensor is placed antero-medial on the distal tibia (1000 Hz), 5 cm above the malleolus, one in each leg. This application has been successfully used in previous investigations, due to the flat bone structure of the tibia at this spot [40, 41].

The reference run includes (1) a standardized warm-up of 800 m at a self-selected speed, (2) followed by three sprinting conditions (one submaximal, two maximal) of 60 m, and (3) an incremental run until the athlete is completely exhausted. The incremental running protocol is developed based on standardized incremental protocols for determining e.g. lactate thresholds [42]. The athletes start the incremental run with a pace of about 2 m/s and increase speed by about 0.3 m/s every 400 m. The duration of the incremental run depends on the athlete's performance, which means it will be finished when the athlete no longer can maintain the predetermined pace of the lap. For the reason of practicability for the athletes, the speed will be controlled with a standardized running watch (Forerunner 245, Garmin, Schaffhausen, Switzerland).

#### Biomechanics laboratory assessment

The biomechanics assessment in the laboratory consists of a 45-min run on an instrumented treadmill (h/p/cosmos sports & medical GmbH, Nussdorf-Traunstein, Germany), with a constant incline of 0.4%, validated to be comparable to outdoor running (Mugele et al. 2018), and several overground running trials. Prior to the protocol, a familiarization will be conducted on the treadmill at a self-selected moderate running speed for five minutes which also serves as a warm-up. Thereafter, another five minutes will be given for joint mobilization and stretching.

Before and after the 45-min treadmill run, 10 overground trials over a level running track with a distance of 10 m will be performed at the same running speed as the treadmill run. Running speed during the overground runs will be recorded using two light barriers (WittyGATE, Microgate Srl, Bolzano, Italy). Subjects will be wearing their preferred running shoes. To quantify the state of fatigue, subjects will be asked to report their rating of perceived exertion according to the Borg scale at 5%, 50% and 95% of the run.

The running speed for the assessments will be set constant and corresponding to 110% of the participants’ average running speed during their continuous training runs with comparable duration over the three months prior to the day of the assessment.

During the trials, kinematic data will be collected using 14 color video cameras (12 × Miqus Hybrid, 2 × Miqus Video, Qualisys AB, Gothenburg, Sweden) at 150 Hz, then it will be processed by the artificial intelligence-based Theia3D motion capture software (Theia Markerless Inc., Kingston, ON, Canada), and further evaluated using Visual3D (C-Motion Inc., Germantown, MD, USA). Additionally, during the treadmill measurements, plantar pressure data will be recorded in sync with the kinematic data with a pressure plate integrated into the treadmill (FDM-T, Zebris Medical GmbH, Weitnau, Germany) at 300 Hz. Data will be recorded over 30 s periods at 5%, 50%, and 95% of the treadmill run total time.

Similarly, during the 20 overground trials, three-dimensional ground reaction forces and moments will be captured employing two force platforms (Advanced Mechanical Technology, Inc., Watertown, USA) at 1200 Hz, synced with the camera system. Five trials with each leg hitting the center of one of the force platforms will be required.

Furthermore, during all trials (instrumented treadmill and overground 10 m track), accelerometer and gyroscope data will be captured with a custom-made inertial sensor system (1000 Hz). The sensors have a dimension of 28 × 45 × 12 mm and will be placed at both feet, at the tibial tuberosities of both legs, at the sacrum, and the region of the xiphoid process with elastic straps (six sensors in total).

From the assessments, relevant kinematic, kinetic, and spatiotemporal parameters will be calculated (Table 1) based on two recent systematic reviews [8, 43] investigating possible biomechanical risk factors for running-related injuries.

**Table 1:** Overview of possible biomechanical risk factor assessment in biomechanics laboratory based on [8, 43]

	Treadmill	Overground
Kinematics	Sagittal ankle angle at footstrike	
	Sagittal knee angle at footstrike	
	Peak ankle dorsiflexion angle	
	Foot strike pattern	
	Peak hip adduction angle	
	Peak ankle eversion velocity	
	Peak ankle eversion angle	
	Peak knee flexion angle	
Kinetics	Vertical plantar peak force (underneath metatarsals II and V)	Vertical ground reaction force peak
	Absolute force–time integral	Vertical impact loading rates
	Anterior–posterior displacement of the center of force	Vertical average loading rates
	Velocity of anterior–posterior displacement	Internal knee abduction moment impulse
	Lateral/medial directed force distribution	Peak external knee adduction
		Vertical impact peak
Temporo-spatial	Cadence	Ground contact time
	Step length	Asymmetry in ground contact time

### Musculoskeletal baseline assessment

The initial clinical assessment is a musculoskeletal and sports medicine examination. As part of this assessment, a blood sample will be taken (< 20 ml) to analyze relevant parameters of bone and muscle status: The biochemical analysis includes hematologic parameters (hemoglobin, erythrocytes, hematocrit, mean corpuscular volume (MCV), mean corpuscular hemoglobin (MCH), mean corpuscular hemoglobin concentration (MCHC), red cell distribution width (RDW), leukocytes, thrombocytes), serum electrolytes (potassium, sodium, chlorine, calcium, phosphate, magnesium), markers of renal function (creatinine, glomerular filtration rate (GFR)), markers of liver function (γ-glutamyl transferase (GGT)), alkaline phosphatase (ALP), creatine kinase (CK), C-reactive protein (CRP), serum electrophoresis (including albumin, α1-, α2-, β-, and ɣ-globulin) (44). Moreover, urinary creatinine excretion is tested. To evaluate additional metabolic or endocrine diseases, thyroid-stimulating hormone (TSH), gastrin, ferritin, vitamin B12, folic acid, parathyroid hormone (PTH), 25-hydroxycholecalciferol (25-OH-D), osteocalcin, procollagen type 1 n-terminal propeptide (P1NP), bone-specific alkaline phosphatase (BAP), serum bone resorption marker carboxy-terminal collagen crosslinks (CTX), as well as the urinary bone resorption marker deoxypyridinoline/crea (DPD) are measured. In addition, pyridoxal-5-phosphate (PLP) levels are evaluated as a potential indicator of a reduced ALP activity [44]. Depending on the clinical examination and skeletal risk profile of the athletes, the physicians will decide on further examinations to assess bone quality (e.g., bone densitometry and/or bone microstructure analysis) [45].

### Continuous data collection during the season

During the season each participant will be equipped with one IMU (SmarTracks Diagnostics DX 5.0, Humotion GmbH, Muenster, Germany) and a belt for application on L5. The IMU should be worn in every training session and during competitions/races. To verify the sensor's data, a training diary will be collected after each run to record the subjective load measured by the Perceptual Wellness Questionnaire [46], and Rating of Perceived Exertion [47] as well as information about training content and conditions (environment, shoes, surface, etc.). Objective load data including distance, time, and velocity will be collected by the athletes’ running watches and the following upload on a social network app (www.strava.com). Injury monitoring is performed by the Oslo Sports Trauma Research Center Questionnaire in its German version [48–50] and will be answered by the athletes once a week. The questionnaire and the training diary will be provided via the app AthleteMonitoring (athletemonitoring.com; FITSTATS Technologies, Inc., Moncton, N-B, Canada).

In case of an injury, athletes will be advised to visit the participating sports medicine physicians for adequate medical diagnosis, possible imaging, and therapy. Furthermore, the above-mentioned biomechanical laboratory assessment will be repeated if possible (determined by mutual decision of participant and sports medicine physicians). To control recovery after the occurrence of an injury the athletes have to answer the University of Wisconsin Running Injury and Recovery Index in its German version [51, 52].

### Outcomes

The primary outcome of the study is the occurrence of injuries to analyze their association to biomechanical, skeletal, or loading parameters by machine learning.

In more detail, the outcome of the study will be presented as:

- Incidence and severity per 1000 exposure hours of overall injuries in competition and training of running athletes.
- Prevalence of nutritive deficits (e.g., vitamin D deficiency) and skeletal alterations. Relationship between clinical baseline assessment and incidence of bone stress injuries during the study period.
- Identification of individual running patterns (stride length, cadence, ground contact time, tibial shock, and tibial acceleration) measured by IMUs and analysis of the relationship between biomechanical running parameters and the incidence of running-related injuries.
- Analysis of further influencing variables (training periodization, subjective stress perception, objective stress, gender, running experience, surface, etc.) on the incidence of running-related injuries.
- Biomechanical changes occurring before and after an injury

### Data processing

All collected data will be anonymized, stored, and saved in the main computer, password-protected. A weekly log will be controlling for new injuries throughout the season. As mentioned, the questionnaires will be available from digital means to the researchers, as well as the clinical personal data.

The synchronized data from the laboratory-based assessments will be processed in Visual3D (C-Motion Inc., Germantown, MD, USA), according to the overground running trials, and the instrumented treadmill run. A Visual3D report will be created based on commonly reported foot strike and toe-off events, including but not limited to kinematic and kinetic data (Table 1). Similarly, IMU data will be processed in Matlab or Pyhthon 3 and a biomechanical report will be created as well.

The reference data from the Humotion IMU will be stored in the SmarTracks Diagnostics DX 5.0 software (Humotion GmbH, Muenster, Germany) on the main computer. Post-processing will be carried out from the 9 channels (accelerometer, magnetometer, and gyroscope) by in-house algorithms from Humotion GmbH.

A master data table will be created with all available variables, categorized by subject and per day/week (continuous IMU season data and daily/weekly questionnaires), including a column to indicate if the subject suffered an injury (injury label).

### Statistical analysis

Data cleaning, feature selection, and validation will be processed, then multivariate analyses and machine learning methods might detect data changes related to an injury. Moreover, demographic and anthropometric data will be processed with descriptive statistics. Characteristics of the population regarding gender or other group variables will be compared using t-tests, Wilcoxon signed-rank tests, X^2^ tests, or Fishers' exact test according to their parametric or non-parametric distribution (injured vs non-injured groups). The statistical analysis will be performed using statistical software R (http://www.R-project.org) or SPSS 25 (SPSS Inc., Chicago, Illinois, USA). The level of significance will be set at *p* < 0.05.

Also, other dimensionality reduction techniques might be employed, such as Principal Component Analysis, to set the weight of certain variables into the machine learning models.

### Machine learning models

**Fig. 2:**
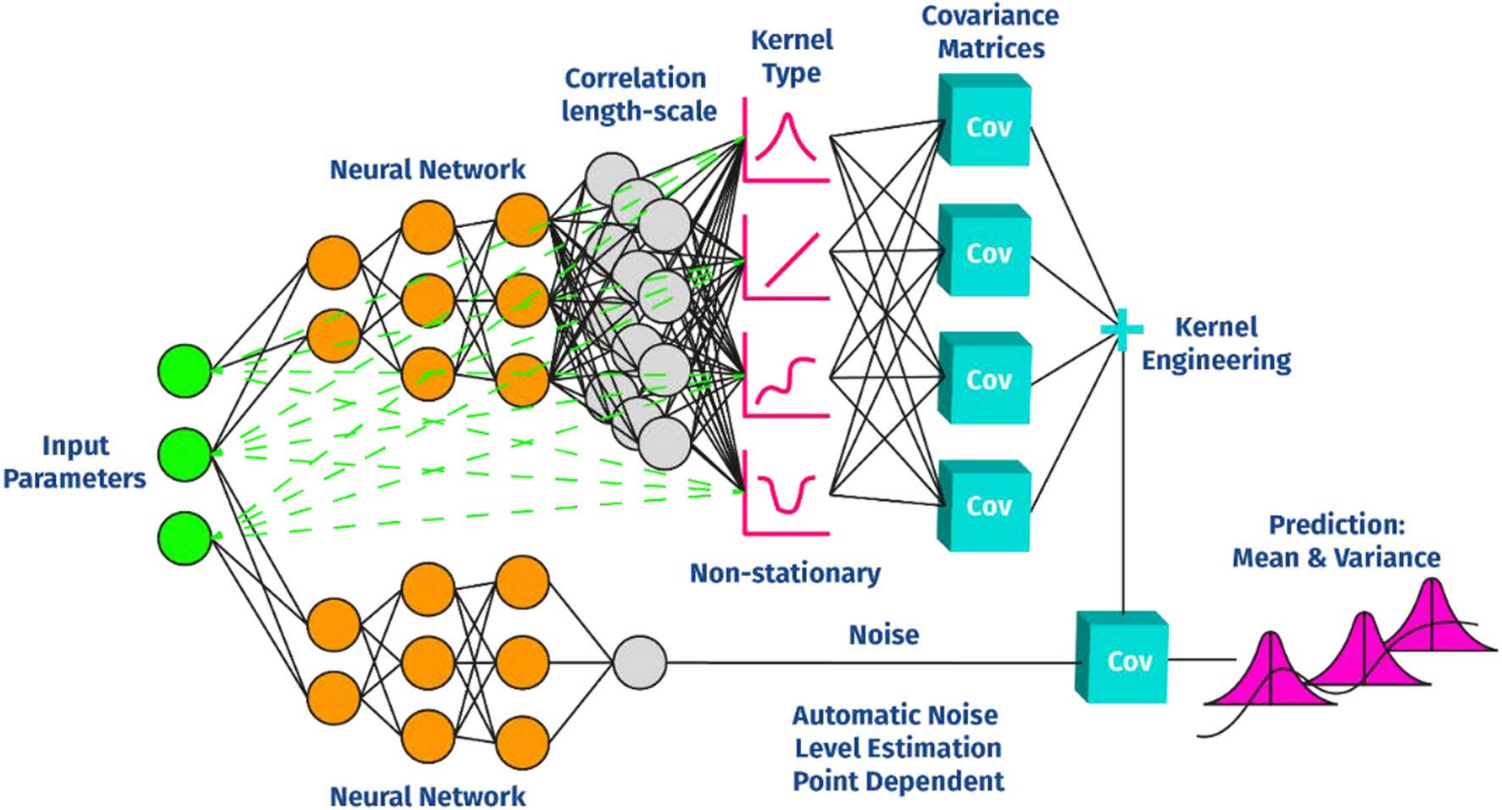
Schematic overview of the DGCN model

The collected data will be analyzed using the probabilistic ML model Deep Gaussian Covariance Network (DGCN). For this purpose, all measured sensor data are used as input parameters $X$ after their processing. The output parameters $Y$ represent for example the ground contact time as well as other variables derived to determine the injury risk. The model learns the functional relationship $Y = \hat{f}\left( X \right) + \in ,$ where $\in$ represents the possible model error. Thereby $\hat{f}\left( X \right)$ is a Gaussian process (${\mathcal{G}\mathcal{P}}$): $\hat{f}\left( X \right)\sim {\mathcal{G}\mathcal{P}}\left( {\mu \left( X \right), K\left( {X,X} \right)} \right)$ with its mean function $\mu \left( X \right)$ and its covariance matrix $K\left( {X,X} \right)$. In the DGCN approach, the free parameters in this model, as well as the covariance matrix, are determined by a coupled neural network such that all free parameters (which must be trained in order to learn the relationship between $X$ and $Y$) are dependent on the data point to predict (see Fig. 2 for a schematic overview of the coupling). This enables the model to represent non-stationary relationships between $X$ and $Y$ in a way that most other stationary methods cannot. For example, when the relationship between $X$ and $Y$ changes due to approaching injuries or different running behaviors such as sprints. In contrast to standard Gaussian processes that can only be applied to a limited number of data points, DGCN can apply the Gaussian process to any number of data points due to its coupling with neural networks. This is possible because batch training can be applied as is common with neural networks. In addition, DGCN allows taking into account the time history of the past data like recurrent neural networks (RNN) can [53].

After the model has been trained, it can be used for new predictions such as injury risk prediction. The already mentioned advantage of a probabilistic ML model is that the uncertainty of the predicted injury risk can also be given, e.g., in the form of a confidence interval. For example, a high predicted injury risk with a wide confidence interval can be less dangerous for the runner than a medium injury risk with a very narrow confidence range. This type of prediction evaluation is not possible with non-probabilistic modeling approaches.

**Fig. 3:**
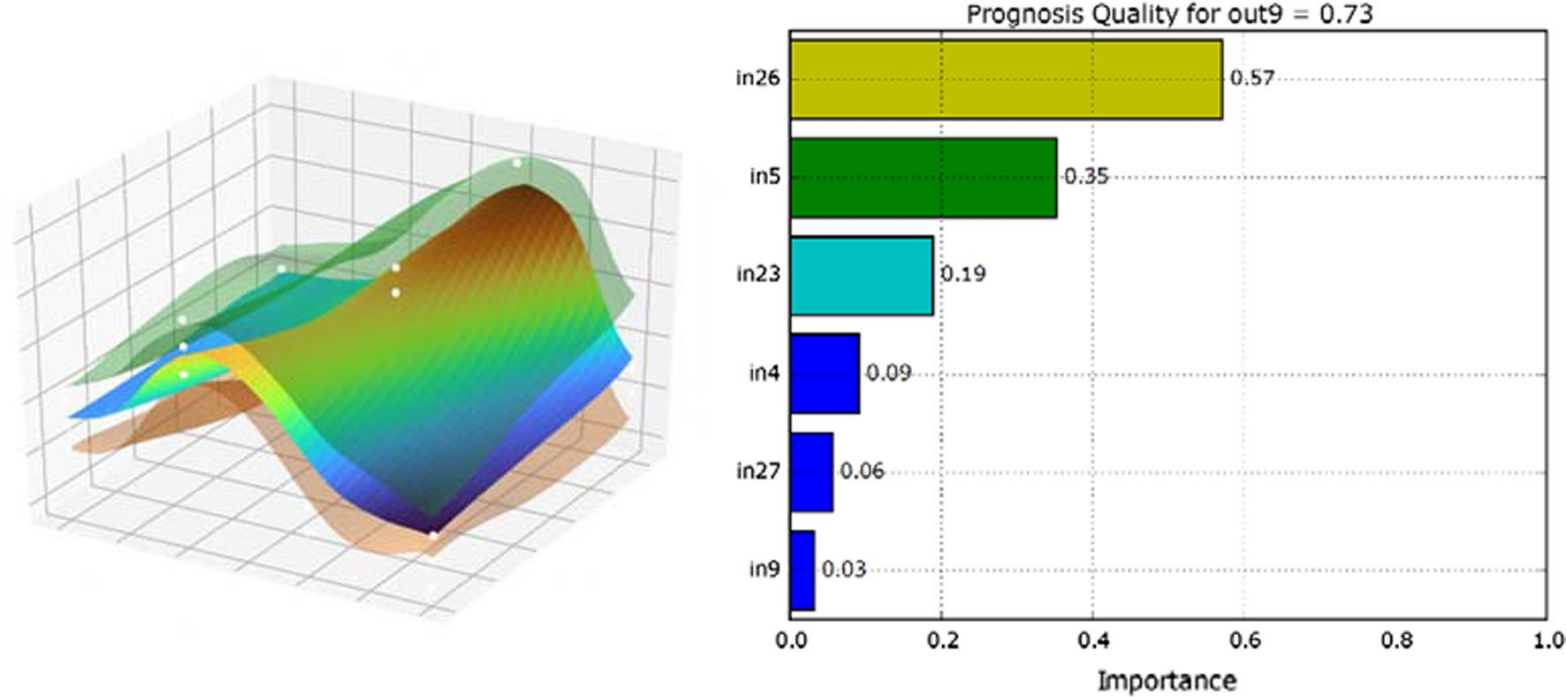
Example for sensitivity analysis

Finally, methods of global variance-based sensitivity [54] analysis will give us a deeper understanding of the learned relationships between the input signals and, for example, the risk of injury. The influence or importance of the input parameters used in the model on the output variable is determined with the help of the model. As a result, ranking of the important parameters is possible as shown in Fig. 3. Also shown in this figure is the trained DGCN model as a function of the two most important input parameters and the output variable to be mapped. The transparent areas represent the 95% confidence interval of the model. Such plots can also provide a deeper understanding of the interdependencies of the parameters.

## Discussion

The study described in this protocol will aim to use prospective injury data collection over a complete season period to (a) assess running injuries and their characteristics, (b) identify and analyze internal and external risk factors as well as identify their interaction, and (c) determine and predict the relationships between internal and external risk factors and running-related injuries using machine learning processes in running athletes.

Considering different running distances and levels, a recent systematic review from 18 prospective studies showed an overall incidence of 40.2% ± 18.8% for running-related musculoskeletal injuries [1]. In over 70% of the running-related injuries, overuse injuries are located at the knee, ankle, lower leg, foot, and toe [1, 55]. For instance, the occurrence of a skeletal overuse injury is related to loading patterns that lead to microdamage and tissue fatigue, and finally to a bone stress injury [56, 57]. The mechanical overloading can develop during multiple sessions (gradual onset) or in a single session (sudden onset) and is dependent upon the structure-specific load capacity [15]. As a result of an imbalance of the mechanical load and the structure-specific load capacity, pathologies such as patellofemoral pain syndrome, plantar fasciopathy, iliotibial band syndrome, bone stress injury, or Achilles tendinopathy can occur [1, 55]. To prevent such pathologies a detailed analysis of the risk factors is necessary. Among others, significant risk factors in running are: previous injuries, higher body mass index, low vitamin D status, impaired bone health [16, 17], higher age, sex, no previous running experience, lower running volume and biomechanical factors [8, 9, 43]. All of these risk factors are in some way attributable to a mismatch of loading and loading capacity.

Thus, an essential component in the analysis of risk factors is the monitoring of internal and external load parameters. In the study presented in this protocol, we will use several standardized methods to monitor the individual internal and external load of each athlete both at baseline and during the study period. One possible way to identify load-dependent consequences at an early stage and to identify further risk factors in running athletes is the monitoring of biomechanical running patterns. Previous systematic reviews indicate that there is some evidence for increased risk due to a greater peak hip adduction [8, 58, 59] and a reduced peak rearfoot eversion in female runners [58]. In a retrospective case–control study, strike patterns and peak vertical ground reaction force were characterized as biomechanical characteristics for some injuries [21]. However, the current literature highlights the need for further research to identify biomechanical factors and their interaction as risk factors in running. Accordingly, one important focus of the present study will be to collect individual biomechanical running parameters by IMUs during every training and competition session and to determine possible changes. These changes can be the result of different initial risk factors such as pre-injury, pain, sex, bone substance, load, environment, the footwear.

Besides biomechanics and cumulative loading parameters, the identification of intrinsic biological risk factors is of major importance. It is well-known that athletes with a reduced tissue-specific loading capacity or inadequate homeostatic regulation following tissue damage are prone to overuse injuries [60–62]. A variety of risk factors such as energy availability, specific nutritional deficits and impaired musculoskeletal tissue quality have been identified for overuse injuries to bone (bone stress injury), tendon (tendinopathy) and muscle (muscle injury), thus further demonstrating the need for a multifactorial approach [6, 60, 61, 63].

To address the multifactorial causation at an interindividual level of risk factors, this study will perform an ML analysis including the discriminative mediators. The advantage of using ML processing is that the model is able to learn from the input data which means, usually ML results in a training phase and a test phase [24]. Feeding ML models with human biomechanical data, especially from IMUs, is already a common practice in activity recognition [64–66], however, the goal in the present study is to input both kinematic and descriptive data to the ML model and to generate injuries as output data to predict the injury risk. Van Eetvelde and colleagues (2021) recently published a systematic review about ML methods to predict and prevent injuries in team sports [23]. The most frequent ML methods used in the included studies were tree-based ensemble methods, Support Vector Machines, and Artificial Neural Networks, resulting in an injury prediction from poor (Accuracy = 52%, AUC = 0.52) to strong (AUC = 0.87, f1-score = 85%) [23]. Based on this systematic review, it can be concluded that the use of ML models for the prediction of risk factors seems to be appropriate.

To the best of our knowledge, no study investigated prospectively the influence of biomechanical, skeletal, and loading risk factors on running-related injuries via machine learning algorithms in running athletes. The results of the planned study may deliver a substantial impact on the early detection of risk factors for running-related injuries. Thus, runners could react to increased risk during training routine by wearing the IMU and using the ML system and actively contribute to minimizing the incidence of injuries in running. Future studies should focus on the system-implemented recommendations in case of an identified increased risk of injury.

## Supplementary Information

**Additional file 1**. Abbreviation list.

## Data Availability

The datasets generated during this study protocol will be available after completion of the study from the corresponding author on reasonable request.

## Declarations

### Study status

The recruitment is still ongoing. It is planned to include 120 subjects into the study.
