# Supplementary material for: A machine learning approach to identify risk factors for running-related injuries: study protocol for a prospective longitudinal cohort trial
Source: BMC Sports Sci Med Rehabil. 2022 Apr 26;14:75. doi: 10.1186/s13102-022-00426-0 (PMC9040327; doi:10.1186/s13102-022-00426-0)
Supplement: Supplementary file 1 — Additional file 1. Abbreviation list. [file 13102_2022_426_MOESM1_ESM.docx]

Additional file 1

Abbreviation list

e.g. For example

BMI Body Mass Index

DPD Desoxypyridinolin

ML Machine Learning

DKRS Deutsche Register Klinischer Studien (German Clinical Trial Register)

DGCN Deep Gaussian Covariance Network

GP Gaussian Processes

SPIRIT Standard Protocol Items: Recommendations for Interventional Trials

CESD-R Center for Epidemiologic Studies Depression Scale-Revised

STAI-Test State-Trait-Anxiety Inventory

RED-S CAT Relative Energy Deficiency in Sport Clinical Assessment Tool

Km Kilometer

IMU Inertial Measurement Unit

GmbH Gesellschaft mit beschränkter Haftung (Company with limited liability)

Hz Hertz

L5 Lumbar Spine 5

Cm Centimeter

M Meter

m/s Meter per second

mm Millimeter

ml Milliliter

MD Maryland

USA United States of America

SPSS Statistical Package for Social Science

RNN Recurrent neural networks

AUC Area Under the ROC Curve

ZIM Zentrales Innovationsprogramm Mittelstand (Central Innovation Program for Medium-sized Companies

KH Karsten Hollander

ALR Anna Lina Rahlf

AS Alberto Sanchez

DF Dominike Fohrmann

JS Julian Stürznickel

TR Tim Rolvin

KC Kevin Cremans

TH Tim Hoenig
